# Exposure to common infections and risk of suicide and self-harm: a longitudinal general population study

**DOI:** 10.1007/s00406-020-01120-3

**Published:** 2020-03-26

**Authors:** Maija Lindgren, Minna Holm, Niina Markkula, Tommi Härkänen, Faith Dickerson, Robert H. Yolken, Jaana Suvisaari

**Affiliations:** 1 Mental Health Unit, Finnish Institute for Health and Welfare (THL), PO Box 30, 00271 Helsinki, Finland; 2 Department of Psychiatry, Helsinki University and Helsinki University Hospital, Helsinki, Finland; 3 Health Monitoring Unit, Finnish Institute for Health and Welfare (THL), Helsinki, Finland; 4 Sheppard Pratt Health System, Stanley Research Program, Baltimore, MD USA; 5 Stanley Division of Developmental Neurovirology, Department of Pediatrics, Johns Hopkins University School of Medicine, Baltimore, MD USA

**Keywords:** *Toxoplasma gondii*, Herpes, Cytomegalovirus (CMV), Epstein–Barr virus (EBV), Herpes simplex virus 1 (HSV-1), Suicide attempt

## Abstract

Common infectious agents, such as *Toxoplasma gondii* (*T. gondii)* and several human herpes viruses, have been linked to increased risk of self-harm. The aim of this study was to investigate the associations between self-harm and seropositivity to *T. gondii*, Epstein-Barr virus (EBV), Herpes Simplex virus Type 1 (HSV-1), and Cytomegalovirus (CMV). IgM and IgG antibodies to these infections were measured in the Health 2000 project nationally representative of the whole Finnish adult population, and 6250 participants, age 30 and over, were followed for 15 years via registers. In addition, lifetime suicidal ideation and suicide attempts based on medical records and interview were assessed within a subsample of 694 participants screened to a substudy for possible psychotic symptoms or as controls. Among the 6250 participants, 14 individuals died of suicide and an additional 4 individuals had a diagnosis of intentional self-harm during follow-up. Serological evidence of lifetime or acute infections was not found to be associated with these suicidal outcomes. However, in the subsample, those seropositive for CMV had fewer suicide attempts compared to those seronegative, adjusting for gender, age, educational level, childhood family size, regional residence, CRP, and screen status (OR for multiple attempts = 0.40, 95% confidence interval 0.20‒0.83, *p* = 0.014). To conclude, common infections were not associated with risk of death by suicide or with self-harm diagnoses at a 15-year follow-up in the general population sample. Our finding of an increased number of suicide attempts among persons seronegative for CMV calls for further research.

**Electronic supplementary material:**

The online version of this article (10.1007/s00406-020-01120-3) contains supplementary material, which is available to authorized users.

## Introduction

The association between mental health and common infection agents has been an increasing area of investigation. The specific infections of interest particularly include *Toxoplasma gondii* (*T. gondii*) and several infections of the herpesvirus family, such as Cytomegalovirus (CMV), Epstein–Barr virus (EBV), and Herpes Simplex virus Type 1 (HSV-1), all infections that primarily often are asymptomatic or cause only mild symptoms, but form a lifelong latent infection and can later reactivate. Immunoglobulin G (IgG) antibodies to these infections indicate lifetime infection, whereas immunoglobulin M (IgM) antibodies may arise during primary infections or reactivation of infection.

*Toxoplasma gondii* is an intracellular parasite, most commonly spread by food or water that is contaminated with oocysts shed by cats, or by eating meat-containing tissue cysts, whereas the herpes viruses spread mostly through bodily fluids, and are among the most common viruses in humans. In recent studies of the general population in Finland, the seropositivity prevalence in adults was 20% for *T. gondii* [1], 72% for HSV-1 [2], 84% for CMV, and 98% for EBV [3]. Associations between these infections and mental health have been repeatedly found. Toxoplasma may be associated with several mental disorders [4–7]. We have previously published cross-sectional results on the association between IgG antibodies against *T. gondii* and anxiety disorders and depressive and psychotic-like symptoms [1, 8]. Furthermore, CMV has been linked to psychiatric disorders [9–12]. Interestingly, in one study, CMV seropositivity influenced mood disorders differentially among males and females, associating with carrying a *lower* risk of mood disorders in males [13]. Also, the association between mental health and HSV-1 and EBV [10, 11, 13–21] has been studied, with somewhat conflicting results.

In addition to mood and emotions, inflammation and infections can also affect behavior [22, 23]. The association between exposure to infectious agents and suicidal behavior has recently raised interest, both in population and clinical studies. Comparing 20 European nations, the prevalence of *T. gondii* was positively correlated with national suicide rates [24], especially in women of postmenopausal age [25]. In a birth-cohort, there was a trend between toxoplasma IgG antibodies and suicide attempts [26] and in a large population cohort, toxoplasma infection was found to be associated with risk of self-directed violence in women [27]. Toxoplasma serointensity has also been associated with suicide attempts in psychiatric outpatients [28] and individuals with mood disorders [29]. However, conflicting results have also been published in schizophrenia patients [30, 31].

In individuals with serious mental illness, those having a lifetime suicide attempt had elevated levels of IgM class antibodies to both *T. gondii* and CMV, and also an association between the levels of these antibodies and the number of suicide attempts was found, whereas IgG class or EBV antibodies were not associated with suicide attempts [32]. In participants with schizophrenia or mood disorders, CMV antibodies were associated with risk of suicide, while HSV-1 and EBV antibodies were not [33]. In another study in the general population, HSV-1 infection was associated with suicidal behavior [34]. A few other studies have not found evidence of association between seropositivity for CMV or HSV-1 and nonfatal suicidal self-directed violence [35] or history of suicide attempt in schizophrenia [36].

One pathway by which infections may influence mental health is inflammation occurring in the central nervous system. The relationship between the inflammatory marker C-reactive protein (CRP) and the studied infections is partly unknown. Higher CRP levels, measuring inflammation, have been reported to be associated with *T. gondii* seropositivity in our previous study [1] and other studies [37, 38] and the role of CRP has also been studied in the context of HSV-1, CMV, and EBV [34, 39, 40]. Inflammation may also trigger changes in affective and behavioral modulation, and inflammatory processes may have a role in suicidality [41].

Information on the possible association on common infections and suicidal outcomes may help prevent self-harm in the population, as the infections may be prevented and treated. In this study, we aimed to investigate whether toxoplasma and herpes infections have a role in the risk for death by suicide or intentional self-harm in the general population. For this purpose, the participants of the Health 2000 study, a large Finnish population survey, were followed-up longitudinally in national health registers for 15 years. As depression is a major risk factor for suicidality [42], we investigated whether being seropositive to the studied infectious agents at baseline was associated with a) baseline depressive symptoms, and b) death by suicide and self-harm during the follow-up.

In addition, we assessed suicidal ideation and suicide attempts retrospectively in a smaller subsample of participants of the Health 2000 study, Psychoses in Finland, selected for possible psychotic symptoms or as controls, and who had been comprehensively assessed using medical records and interview data [43].

## Methods

### Study procedure and measuring of antibodies

Data from the Health 2000 (BRIF8901), a nationally representative survey of the Finnish general population [44], were used. Altogether 8028 persons, aged 30 years and over, were sampled using stratified two-staged cluster sampling, with participants representing their age and gender group in the area where they lived. All participants gave written informed consent and the study was approved by the ethics committees of the Hospital District of Helsinki and Uusimaa and the Finnish Institute for Health and Welfare. The study was carried out in accordance with the Declaration of Helsinki.

The baseline study was conducted in 2000–2001. IgG antibodies to *T. gondii*, HSV-1, EBV, and CMV were measured at baseline from plasma samples of 6250 participants. CRP levels were analyzed from serum samples, taken at the same time as plasma samples. Beck Depressive Inventory (BDI, with 21 items) was completed at baseline by 5786 (72%) of the participants, assessing current depressive symptoms [45]. IgM antibodies to *T. gondii* and CMV were measured from two subsamples, people who had participated in a substudy on psychotic disorders and symptoms (see “Psychoses in Finland substudy” section) and a matched case–control study of those who had attempted or committed suicide in the follow-up (see “15-year register follow-up” section). By assessing both IgG and IgM markers, we hoped to differentiate recent infections from those that occurred years earlier.

The analyses were performed by solid-phase enzyme immunoassay as previously described [46]. The participants were rated seropositive for herpes viruses if their optimal density value was above or at the infection point in the sample distribution. 50 IU/ml was used as the cut-off for toxoplasma IgG seropositivity. The IgM values are expressed as a ratio of the raw value to a control run on each plate as well as plate adjusted to a mean = 2 and standard deviation (SD) = 1, where a value of 3 or greater represented 1 SD greater than the mean of the population and was used as the cutoff point for seropositivity. In addition, serointensity, defined as the quantitative level of antibody in terms of standardized units, was used as a continuous variable.

### 15-year register follow-up

Two registers were used to gather follow-up data on the total participant group of 6250 persons. Data on suicide deaths was available until the end of the year 2015 from the Causes of Death statistics (Statistics Finland), yielding a register follow-up time of approximately 15 years. The register contains information on date and cause of all deaths in Finland. Second, data on intentional self-harm were acquired from the Finnish hospital discharge database, a part of the Care Registration for Health Care (HILMO) up to the end of 2015, consisting of data from both inpatient and outpatient settings. The intentional self-harm outcome included ICD-10 diagnoses X60–X84, Y87.0, Z72.8, and Z91.5.

### Psychoses in Finland substudy

**Fig. 1:**
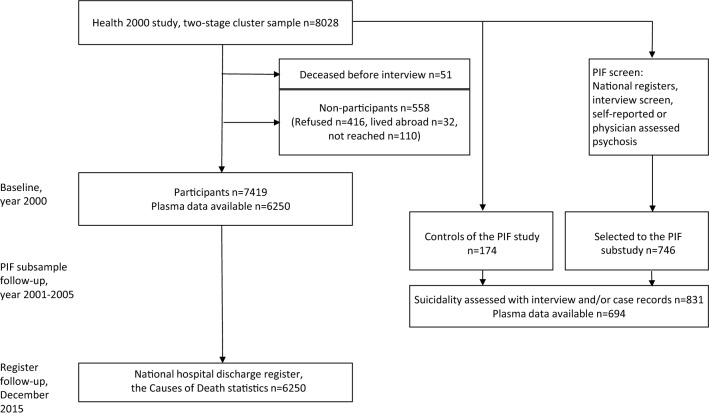
The participants of the register follow-up and the PIF subsample

Between December 2001 and January 2005, a subsample of the Health 2000 survey participants took part in the Psychoses in Finland (PIF) substudy [47]. A total of 746 participants were screened to this substudy based on suspected psychosis, either based on baseline general population study or national registers (Fig. 1). All participants gave written informed consent. People with hospital treatment or medication for psychosis were included in the PIF study, as were individuals on disability pension because of a severe mental disorder, which could be a depressive disorder. The PIF participants also included 174 control subjects assessed for the lifetime occurrence of suicidal ideation and behavior. The interval from the time of the Health 2000 main study, including blood samples, to the interview about suicide behavior was 1.0‒4.3 years (mean 2.2, SD 0.7 years). Additionally, medical records from lifetime mental health treatment contacts were reviewed, including those of participants selected to the PIF study who had not been interviewed. A best estimate for suicidal thoughts and acts was made by experienced psychiatrists based on all available systematically evaluated information, including self-reported information, medical records, and health care registers [43]. The participants in this group consisted of 831 individuals, of whom 54.5% were female.

### Data analyses

Analyses of the total sample were conducted taking into account the two-stage cluster sampling design using the survey package [48] of R. In addition, post-stratification weights were used to adjust for the oversampling of individuals aged 80 years and over and non-response, the results thus representing the whole Finnish adult population. As post hoc analyses, we ran the same analyses among those seropositive for each infection, using antibody levels to predict the depressive symptoms or suicide/self-harm. The significant analyses were repeated in females and males separately because of the previous gender-specific results.

#### Depression and infections

Cross-sectional associations of baseline BDI scores and seropositivity/antibody levels were calculated with linear regression models adjusting for background variables associated with infection seropositivity: gender, age, educational level, region of residence, and self-reported number of siblings (as a proxy for childhood family size). All IgG analyses were also adjusted for the plate used in the assays to account for a platform effect in running the assays. Of the linear models, unstandardized Beta values are reported.

#### 15-year register follow-up

In the 15-year register follow-up, IgG levels were available from the whole sample at baseline. For IgM levels, a case–control approach was used: for each case (individual who had either died by suicide or had a self-harm diagnosis during the follow-up), the goal was to find 5 controls of the same age (± 2 years), gender, and health center district. Altogether 73 eligible matched controls were chosen (1‒5 per case, mean 4.1, median 5).

Cox regression analyses predicting suicide/self-harm were conducted, with seropositivity for the chosen infections as the main predictor, while again adjusting for the same background variables. Hazard ratios (HR) are reported for these models.

#### Psychoses in Finland substudy

The PIF subsample analyses were conducted using IBM SPSS Statistics for Windows, version 25. We used binary logistic regression to investigate whether the seropositivity/antibody levels were associated with suicidal thoughts, and multinomial logistic regression to investigate whether they were associated with single or multiple suicide attempts, the reference category being No suicide attempts. Odds ratios (OR) are reported of the logistic models.

We controlled for gender, age, educational level, region of residence, number of siblings, screen status, and, at the last phase, CRP (as a logarithmic transformation), as we wanted to investigate whether the possible associations were explained by low-grade infection.

## Results

The characteristics of the sample and the prevalences of the antibodies in the analysis groups can be seen in Table 1. We have previously reported that CRP was associated with IgG antibodies for *T. gondii* [1]. Adjusted for age and sex, higher CRP levels were also associated with seropositivity for HSV-1 IgG antibodies (*p* < 0.001) but not with other antibodies. CRP and BDI scores were negatively associated (*p* < 0.001).

**Table 1:** Baseline characteristics and prevalences of the antibodies in the analysis groups

	Total sample, *n* = 6250^a^	PIF subsample *n* = 694^b^	Cases, *n* = 18^b,c^	Matched controls, *n* = 73^b,c^	Cox model, HR (95% CI), *p* value^d^
Females	52.2 (50.9‒54.0) %	370 (53.3%)	4 (22.2%)	20 (27.4%)	–
Age	52.1 (51.7‒52.5)	52.7 (14.1), 30‒90	48.1 (12.2), 30‒71	49.1 (10.9), 30‒73	–
Basic education only	40.8 (39.4‒42.0) %	291 (41.9%)	3 (16.7%)	23 (31.5%)	–
Number of siblings	3.8 (3.7‒3.8)	3.9 (2.9), 0‒18	3.7 (3.1), 0‒10	3.5 (2.8), 0‒14	–
Toxo IgG seropositivity	19.7 (18.3‒21.0) %	172 (24.8%)	4 (22.2%)	15 (20.5%)	1.91 (0.63, 5.76), 0.250
Toxo IgG antibody level	27.3 (26.1‒28.5)	31.0 (31.3), 3.4‒179.4	29.2 (30.6), 8.1‒88.4	23.6 (25.4), 4.4‒120.7	1.01 (1.00, 1.02), 0.128
Toxo IgM seropositivity	N/A	119 (17.1%)	4 (25.0%)	13 (18.3%)	1.38 (0.37, 5.10), 0.634
Toxo IgM antibody level	N/A	2.0 (1.0), − 0.1 to 5.0	2.1 (1.0), 0.8‒4.0	1.9 (1.0), 0.4‒4.4	1.14 (0.66, 1.99), 0.643
CMV IgG seropositivity	83.5 (82.5‒84.0) %	583 (84.0%)	15 (83.3%)	59 (80.8%)	1.03 (0.30, 3.57), 0.957
CMV IgG antibody level	4.2 (4.1‒4.3)	4.2 (2.2), 0.1‒9.9	3.4 (1.7), 0.4‒5.7	3.6 (2.0), 0.3‒7.6	0.89 (0.74, 1.07), 0.238
CMV IgM seropositivity	N/A	114 (16.4%)	2 (12.5%)	6 (8.5%)	1.24 (0.18, 8.79), 0.828
CMV IgM antibody level	N/A	2.0 (1.0), 0.7‒6.1	1.8 (0.7), 0.8‒3.2	1.8 (0.8), 0.8‒4.2	0.88 (0.41, 1.89), 0.750
EBV IgG seropositivity	97.9 (97.5‒98.0) %	678 (97.7%)	18 (100%)	73 (100%)	N/A
EBV IgG antibody level	1.5 (1.4‒1.5)	1.4 (0.7), 0.1‒4.5	1.5 (0.9), 0.4‒3.6	1.5 (0.7), 0.4‒3.7	0.98 (0.49, 1.96), 0.964
HSV-1 IgG seropositivity	71.2 (70.0‒72.0) %	496 (71.5%)	14 (77.8%)	47 (64.4%)	1.69 (0.55, 5.22), 0.360
HSV-1 IgG antibody level	3.7 (3.7‒3.8)	3.8 (2.8), 0.1‒16.9	3.8 (2.5), 0.2‒8.5	3.7 (3.3), 0.2‒12.7	1.01 (0.88, 1.15), 0.942

*HR* Hazard Ratio (95% CI, confidence interval), *p* value for the infection variable

^a^Calculated with weights to adjust for the oversampling of individuals aged ≥ 80 years and non-response. Prevalences (95% CI), or mean (95% CI). Only IgG levels available from the total sample

^b^*N* (%), or mean (SD), range

^c^Suicide or self-harm diagnosis during the 15-year register follow-up (cases), and the control subjects for them, matched for age, gender, and health center district

^d^Cox regression model predicting case status; adjusting for gender, age, educational level, region of residence, and number of siblings

### Cross-sectional associations of baseline depression and herpes infections

We used linear regression models controlling for gender, age, educational level, region of residence, and number of siblings to predict BDI scores, measuring self-reported depressive symptoms. Besides the previously reported association with *T. gondii* [1], we found no significant associations with the herpes infections (Table 2).

**Table 2:** Linear regression models predicting baseline depressive symptoms (BDI score)

	Beta value	*p* value
Toxoplasma IgG seropositivity	**0.51**	**0.035**
Toxoplasma IgG antibody level	0.01	0.060
Toxoplasma IgM seropositivity	0.80	0.421
Toxoplasma IgM antibody level	− 0.23	0.546
CMV IgG seropositivity	0.12	0.631
CMV IgG antibody level	0.02	0.588
CMV IgM seropositivity	0.75	0.469
CMV IgM antibody level	0.17	0.669
EBV IgG seropositivity	0.39	0.479
EBV IgG antibody level	− 0.06	0.667
HSV-1 IgG seropositivity	0.11	0.600
HSV-1 IgG antibody level	0.016	0.631

All models adjusting for gender, age, educational level, region of residence, number of siblings, and IgG analyses also for plate

Association with IgG levels, *n* = 5786, weighted to adjust for the oversampling of individuals aged ≥ 80 years and non-response. Association with IgM levels, *n* = 705

Significant values in bold

Female gender, older age, and low educational level remained as significant correlates of depressive symptoms in the models. Both depressive symptoms and seropositivity/antibody levels to the infections increased with age, the association between depressive symptoms and age being relatively linear. Using age as a quadratic term did not change the results. Given the non-normal distribution of the BDI score, we also tried ordinal regression and Tobit regression models, with similar results.

The analyses were repeated using serointensity among those seropositive for each infection, and there were no significant associations between serointensities and BDI score.

### 15-year register follow-up

Of the participants, 1629 of the original 8028 (20.3%) had died during the follow-up period; 15 (0.2%) of the deaths were recorded as suicides, and 14 of these individuals had given plasma samples at baseline in 2000. Two of the suicide deaths were females and the other 12 were males. In addition, four individuals (two males and two females) had a treatment episode recorded with a diagnosis of intentional self-harm (Z91.5, history of self-harm or Z72.8, self-damaging behavior), after year 2000. Altogether 18 individuals who had either died by suicide or had a self-harm diagnosis during the follow-up and who had given a blood sample where thus defined as cases. In Cox regression models, male gender and Western and Northern regions of residence in Finland were associated with the case status, while none of the IgG or IgM seropositivity/antibody levels of any of the infections or CRP were significant predictors (Table 1).

We next ran the same Cox analyses using serointensity among those seropositive for each infection. Among those seropositive for CMV, higher CMV IgG serointensity predicted lower risk of self-harm (HR = 0.62, 95% CI 0.43‒0.91, *p* = 0.014), when adjusting for gender, age, number of siblings, region of residence, and education. Other serointensity variables were not predictive of self-harm among those seropositive for that infection.

### Psychoses in Finland subsample

Of the PIF participants, 694 of 831 had given plasma data at baseline, of whom 129 (18.6%) were included as control subjects (screen-negatives) and the rest were screened to the substudy based on self-reported or register-based suspicion of psychosis (screen-positives). According to the Structured Clinical Interview for DSM-IV (SCID-I) [49] and information from all medical records from lifetime mental health treatment contacts, 167 (24.1%) of the 694 participants in the subsample were diagnosed with any lifetime DSM-IV psychotic disorder and 191 (27.5%) had a mood disorder without a psychotic disorder. Of the rest, 120 (17.3%) had some other lifetime psychiatric diagnosis and 206 (29.7%) had no psychiatric diagnosis.

#### Suicidal thoughts in the subsample

A total of 218 of 694 (31.4%) had ever had suicidal thoughts based on interview and medical records. Suicidal thoughts were as common in males as in females and were significantly associated with psychotic (*p* < 0.001) and mood disorder (*p* < 0.001) diagnoses and with entering the study as screen-positive (*p* < 0.001) but not with CRP (*p* = 0.525). There were no significant associations between suicidal thoughts and infection seropositivity/antibody levels (Table 3).

**Table 3:** Logistic regression models predicting lifetime suicidal thoughts and attempts in the PIF subsample (*n* = 694)

	Suicidal thoughts (no/yes)		Suicide attempts (none/one/multiple)	
	OR (95% CI)^a^	*p* value	OR (95% CI)^b^	*p* value
Toxo IgG seropositivity	0.76 (0.50, 1.16)	0.196	0.98 (0.47, 2.08)	0.966
Toxo IgG antibody level	1.00 (0.99, 1.00)	0.125	1.00 (0.99, 1.01)	0.941
Toxo IgM seropositivity	1.06 (0.67, 1.67)	0.815	0.91 (0.38, 2.16)	0.828
Toxo IgM antibody level	1.08 (0.91, 1.29)	0.379	1.03 (0.74, 1.44)	0.846
CMV IgG seropositivity	0.85 (0.53, 1.37)	0.511	**0.45 (0.22, 0.91)**	**0.026**
CMV IgG antibody level	0.99 (0.91, 1.08)	0.796	0.92 (0.80, 1.07)	0.289
CMV IgM seropositivity	0.75 (0.46, 1.24)	0.261	0.61 (0.22, 1.68)	0.342
CMV IgM antibody level	0.97 (0.81, 1.16)	0.713	0.87 (0.62, 1.22)	0.413
EBV IgG seropositivity	0.60 (0.19, 1.83)	0.365	0.46 (0.92, 2.33)	0.350
EBV IgG antibody level	1.01 (0.76, 1.33)	0.958	1.18 (0.72, 1.92)	0.518
HSV-1 IgG seropositivity	1.15 (0.77, 1.73)	0.485	0.88 (0.44, 1.75)	0.716
HSV-1 IgG antibody level	1.00 (0.94, 1.07)	0.968	0.94 (0.83, 1.06)	0.331

All models adjusting for gender, age, educational level, region of residence, number of siblings, screen status, and IgG analyses also for plate. Significant values in bold

*CI* confidence interval, *OR* odds ratio of the seropositivity or antibody level

^a^OR for suicidal thoughts, the reference category is No suicidal thoughts

^b^OR for multiple attempts, the reference category is No suicide attempts

#### Suicide attempts in the subsample

A total of 90 of 694 (13.0%) of the subsample had a history of a suicide attempt, 42 (6.1%) once and 48 (6.9%) multiple times, with no significant gender differences. Of those with a history of a suicide attempt, 54 (60.0%) had a psychosis diagnosis and 29 (32.2%) had a mood disorder without a psychotic disorder. Suicide attempts were associated with psychotic (*p* < 0.001) and mood disorder (*p* < 0.001) diagnoses, with entering the study as screen-positive (*p* = 0.007), and with higher CRP (*p* = 0.004).

In multinomial logistic regression models adjusting for gender, age, education, number of siblings, region of residence, and screen status (Table 3), CMV IgG seropositivity was associated with fewer suicide attempts (OR for multiple attempts = 0.45, 95% confidence interval (CI) 0.22‒0.91, *p* = 0.026). When CRP was also controlled for, CMV seropositivity remained a significant predictor (OR = 0.40, 95% CI 0.20‒0.83, *p* = 0.014; Supplementary Table 4). Further post hoc analyses revealed that the association between CMV seropositivity and history of a suicide attempt was significant among the persons with a mood disorder (OR for multiple attempts = 0.17, 95% CI 0.05‒0.58, *p* = 0.005) but not among those with a psychotic disorder (*p* = 0.313) or the participants with neither diagnosis (*p* = N/A).

In post hoc analyses looking at males and females separately, there were significant associations only in males: seropositivity for CMV was again associated with fewer suicide attempts (OR for multiple attempts = 0.26, 95% CI 0.10‒0.69, *p* = 0.006); controlling also for CRP did not change the result (OR = 0.26, 95% CI 0.10‒0.71, *p* = 0.008). Lower CMV IgG antibody levels were significantly associated with males’ multiple suicide attempts also using the intensity of antibodies (OR = 0.77, 95% CI 0.62‒0.96, *p* = 0.022), also when controlling for CRP (OR = 0.77, 95% CI 0.61‒0.97, *p* = 0.027). In addition, in males, EBV serointensity was associated with suicide attempts (OR for one attempt versus no attempts = 2.12, 95% CI 1.12‒4.02, *p* = 0.022). EBV remained a significant predictor even when controlling for CRP (OR = 2.13, 95% CI 1.11‒4.09, *p* = 0.024; Supplementary Table 5).

## Discussion

First, we found no significant cross-sectional associations between baseline depressive symptoms and antibody levels to the herpes viruses EBV, CMV, or HSV-1 when controlling for background variables associated with infection seropositivity. We have previously reported an association between *T. gondii* and depressive symptoms in this sample, even when adjusting for age, gender, region of residence, education, marital status, cat ownership, 12-month diagnoses, CRP, and antidepressant use [1]. Some previous studies have found CMV but not HSV-1 to be linked with depression [11, 14, 50], but the association between exposure to herpes infections and depressive symptoms has not been extensively studied.

Second, in a general population cohort of 6250 participants and 15-year follow-up using comprehensive register data and complete follow-up, seropositivity or antibody levels of *T. gondii* or herpes viruses were not found to be associated with suicides or diagnoses of intentional self-harm.

Third, we assessed suicidal ideation and suicide attempts in a subsample using information from medical records as well as data collected by questionnaire and interview. The participants of this subsample had either a severe psychiatric disorder or were controls; we controlled for this screen status in the regression models. A total of 31% of this subsample had had suicidal thoughts and 13% had attempted suicide during their lifetime. EBV antibody level was associated with a history of a suicide attempt in males, and none of the other infection variables added to the association with the suicide measures. However, those seropositive for IgG class antibodies for CMV, measuring latent infection, had fewer suicide attempts compared to those who were seronegative. This result was especially significant among male participants and also high CMV serointensity showed the same protective effect. The association was specific to CMV, as antibodies to the other studied herpes infections did not show the same relationship, and the association with EBV was in the opposite direction.

Converging evidence suggests that some common infectious agents may predispose to mental disorders and disrupt affective and behavioral modulation [6, 12, 27, 51], hence also possibly elevating risk of self-harm. Only few prospective studies have been conducted [27, 33] and there are differences between study design, seropositivity cut-offs used, populations studied, variables controlled for, and the timelines, which can all cause discrepancies between studies. Most of the previous studies have been conducted in clinical samples, only few studies concentrating on suicidality in the general population [26, 27]. Previous studies have reported self-harm to be associated with toxoplasma infection in the population especially in women [25, 27]. In psychiatric samples, antibodies for toxoplasma have been positively associated with suicidality [28, 29, 32, 36, 52], however, not all studies have found such association [30, 31]. The differences between the current study and a similarly prospective study by Pedersen et al. [27] include their larger sample and a broader definition of the suicidal outcomes. The current study found no association between *T. gondii* antibodies and the suicidal outcomes of interest when controlling for the confounding variables, although *T. gondii* was associated with depressive symptoms [1].

The association between herpes viruses and suicidality has been studied scarcely [32–36] and mostly among psychiatric samples. In previous works, elevated CMV antibodies have been associated with suicide attempts or death by suicide in some psychiatric samples [32, 33] but not in all [36]. In a prospective study where elevated levels of CMV antibodies predicted suicide [33], the participants were mostly schizophrenia or bipolar disorder patients. In our study, we had the reverse result of lower CMV antibodies associating with multiple suicide attempts, but the association was not significant among those with a psychotic disorder. In another previous work, IgG class antibodies against HSV-1 were associated with attempting or committing suicide in the general population [34]. A few previous studies have reported negative findings on the association between suicidality and EBV or HSV-1 [32, 33, 35, 36]; to our knowledge, our finding that higher EBV antibodies were associated with risk of a lifetime suicide attempt is new. In a previous longitudinal study, EBV antibody levels were stable at the individual level but there was strong variation among individuals [53]. As overall EBV antibody level was measured here, the results could look different if we would measure specific EBV proteins, as was done by Dickerson and colleagues [20].

Although the prevalence of CMV in Finland has decreased significantly during recent decades [54], the rate was as high as 84% in the population at our baseline in year 2000 [3]. The increased odds of multiple suicide attempts in persons belonging to the CMV seronegative minority is a new finding. In line with this was our post hoc result of higher CMV antibodies predicting lower risk of self-harm in the register follow-up among those seropositive for CMV. In the same sample as used in the current study, we have also found CMV to protect from new-onset generalized anxiety disorder, but not from new-onset depressive disorders or other anxiety disorders [55]. Low levels of CMV antibodies in affective disorders have also been found in other studies (Yolken et al., unpublished results). One previous study found that CMV seropositivity associated with higher risk of mood disorders in females, but—in line with our results—with a lower risk in males [13]. The association between the immune system and mental health may be different in females and in males [13, 56, 57], although the reasons for this are still unclear, leading to a question whether gender differences may exist in the effect of infections on suicidality. As transmission of CMV requires intimate contact with other people, one could speculate whether persons seronegative for CMV have personality factors that predispose to suicidality as well, such as neuroticism [58] or social isolation. However, HSV-1 and EBV have similar modes of person-to-person transmission as CMV, and our result was specific to CMV.

Attempting and dying by suicide may be associated with infections in different ways. Furthermore, we found that CMV seronegativity only added to the risk for several attempts but not to the risk for one attempt, when compared to no suicide attempts. Those attempting suicide once or several times can be clinically different populations, and the risk factors for single or repeated suicide attempts can be different [59].

Lower socioeconomic status is associated with seropositivity for CMV [60, 61]. In the general population, suicide risk is also associated with lower socioeconomic status [62, 63]. Therefore, it seems surprising that antibodies for CMV were associated with lowered suicide risk. However, among patients treated for major depressive disorder, the association between socioeconomic status and suicidality has been the opposite in Finland: higher educational level and family income predict suicide mortality [64]. In another Finnish study investigating people with depression-based disability retirement, high socioeconomic position did not protect against unnatural and alcohol-related deaths, unlike in the general population [65]. Our result of the protective role of CMV was significant especially among those with mood disorders. It was not significant when only looking at those with psychotic disorder in the PIF subsample, and suicide attempts were not associated with educational level among those with a psychotic disorder in the PIF study [43]. In other words, the interplay among socioeconomic status, psychiatric symptoms, and infections in regards to suicide risk seems to be complex.

Another factor to consider is general inflammation. Inflammatory processes may be linked to suicidality, and increased CRP levels are associated with mortality risk in people with mental disorders [66, 67]. The associations found in the PIF subsample remained when adjusting for CRP, so they were not explained by inflammation. Another reason why general activation of the immune system is not likely to explain our findings was that they were specific to certain infections.

### Strengths and weaknesses

A number of limitations should be kept in mind when evaluating the current results. Two samples were used in the study, both having their strengths and weaknesses. In the large general population of sample representative of the whole adult population in Finland, we were able to prospectively investigate the role of serological factors in suicide deaths and self-harm. Suicide deaths were reliably defined in the Causes of Death statistics, although the rareness of the outcome severely limited the statistical power of the analyses. Power calculations show that with only 18 cases, to have a 60% power, a HR as high as 3–4 would be needed. Only the most severe forms of intentional self-harm were captured in the health care register follow-up, and cases of self-harm not resulting in medical care or not diagnosed as self-harm were missed. Furthermore, IgM antibodies were only available for cases and matched controls and for the PIF subsample, while the whole sample could be used when investigating IgG levels. Furthermore, IgM levels informed whether the infection had occurred close to the baseline assessment, but we did not have information on IgM levels at the time of self-harm or suicide.

Using the smaller PIF subsample, we were able to assess suicidal ideation and suicide attempts more reliably, as conclusive, retrospective information from self-report, medical records, and health care registers was available. Persons with severe mental illness were enriched in this subsample, limiting the generalizability of these results to the general population, which is why we controlled for screen status and also looked at this subsample dividing it based on diagnosis group. The subsample was screened using multiple sources of information and included psychosis patients and individuals with any suspicion of psychotic illness as well as about 20% of matched healthy controls. One of the screens was disability pension based on a severe mental disorder (often depressive disorder) and suicidality was thus common in the subsample. In the subsample, lifetime suicidality was assessed, so the suicidal thoughts or acts may have happened before the blood sample was taken in year 2000. These kinds of differences in study design might explain some of the discrepancies noted among past studies. Genders were investigated separately in post hoc analyses but the small sample sizes limit the value of these findings, especially in females having small cell numbers in some analyses.

In analyzing the associations between infections and self-harm, we adjusted for various background variables related to the measured infections, including demographic factors and inflammation. We did not assess for all the factors that could contribute to suicide such as trauma history, psychiatric symptoms, substance use, or personality factors [68]. Finally, multiple comparisons were not corrected for in this exploratory study. If the false discovery rate had been controlled for using the Benjamini–Hochberg procedure [69], the associations would have not remained significant.

### Conclusions

In a large sample representing the whole Finnish adult population, antibodies to CMV, EBV, or HSV-1 were not associated with depressive symptoms. Antibodies to *T. gondii* or the herpes viruses were not associated with heightened risk for subsequent suicide deaths or diagnoses of intentional self-harm in a 15-year register follow-up. In a subsample consisting mostly of participants with severe mental disorders, the infections were not associated with a heightened risk for suicidal thoughts or acts. However, the finding of heightened suicidality risk among persons not infected with CMV calls for further research.

## Electronic supplementary material

Below is the link to the electronic supplementary material.

Supplementary file1 (PDF 336 kb)
