# Supplementary material for: Exposure to common infections and risk of suicide and self-harm: a longitudinal general population study
Source: Eur Arch Psychiatry Clin Neurosci. 2020 Mar 26;270(7):829–39. doi: 10.1007/s00406-020-01120-3 (PMC7474710; doi:10.1007/s00406-020-01120-3)
Supplement: Supplementary file 1 — Supplementary file1 (PDF 336 kb) [file 406_2020_1120_MOESM1_ESM.pdf]

Supplementary material

Lindgren et al. Exposure to common infections and risk of suicide and self-harm – a longitudinal general population study

Table 4. Multinomial logistic regression model of lifetime suicide attempts in the PIF subsample (n=694); all variables entered in the model simultaneously. The reference category is No suicide attempts.

	One suicide attempt				Multiple suicide attempts			
	OR	95% CI for OR		Sig.	OR	95% CI for OR		Sig.
		Lower	Upper			Lower	Upper	
Gender								
Male	1.30	0.67	2.56	0.440	0.91	0.48	1.73	0.769
Female								
Age	0.99	0.96	1.02	0.584	**0.94**	**0.92**	**0.97**	**<0.001**
Education								
Basic	0.59	0.25	1.38	0.224	**6.44**	**2.30**	**18.02**	**<0.001**
Secondary	0.76	0.33	1.73	0.508	**2.92**	**1.02**	**8.41**	**0.047**
High								
Number of siblings	0.87	0.75	1.01	0.067	0.91	0.80	1.04	0.182
Region of residence in Finland								
Southern	0.49	0.16	1.50	0.209	0.80	0.31	2.09	0.656
Southwestern	0.51	0.12	2.19	0.366	0.90	0.27	3.04	0.861
Western	1.29	0.42	3.95	0.656	1.11	0.38	3.24	0.848
Eastern	1.87	0.66	5.31	0.242	1.18	0.41	3.40	0.758
Northern								
Plate used in the assays								
Plate 0	0.94	0.47	1.90	0.863	1.07	0.53	2.13	0.859
Plate 1								
Screen status								
Screen-positive: suspicion of severe mental disorder	3.26	0.97	11.01	0.057	**4.27**	**1.26**	**14.45**	**0.020**
Screen-negative: controls								
C-reactive protein	**1.34**	**1.09**	**1.64**	**0.006**	**1.33**	**1.10**	**1.62**	**0.004**
CMV IgG seroprevalence								
Positive	2.24	0.65	7.75	0.202	**0.40**	**0.20**	**0.83**	**0.014**
Negative								

Significant values in bold.
OR, odds ratio
CI, confidence interval

Table 5. Multinomial logistic regression model of lifetime suicide attempts in males in the PIF subsample (n=309); all variables entered in the model simultaneously. The reference category is No suicide attempts.

	One suicide attempt				Multiple suicide attempts			
	OR	95% CI for OR		Sig.	OR	95% CI for OR		Sig.
		Lower	Upper			Lower	Upper	
Age	0.99	0.95	1.03	0.532	**0.90**	**0.86**	**0.95**	**<0.001**
Education								
Basic	1.32	0.39	4.43	0.657	**9.16**	**1.78**	**47.08**	**0.008**
Secondary	0.77	0.21	2.92	0.705	2.77	0.52	14.60	0.231
High								
Number of siblings	0.87	0.71	1.07	0.189	1.01	0.84	1.22	0.911
Region of residence in Finland								
Southern	0.42	0.08	2.28	0.316	1.01	0.29	3.51	0.989
Southwestern	0.52	0.08	3.56	0.508	0.27	0.03	2.66	0.262
Western	1.74	0.38	7.93	0.473	0.85	0.19	3.80	0.826
Eastern	2.40	0.55	10.43	0.244	1.42	0.34	6.02	0.633
Northern								
Plate used in the assays								
Plate 0	1.61	0.52	4.94	0.406	0.76	0.27	2.13	0.607
Plate 1								
Screen status								
Screen-positive: suspicion of severe mental disorder	2.16	0.57	8.17	0.256	4.06	0.83	19.89	0.083
Screen-negative: controls								
C-reactive protein	1.24	0.94	1.64	0.134	**1.40**	**1.07**	**1.84**	**0.015**
EBV IgG antibody level	**2.13**	**1.11**	**4.09**	**0.024**	1.59	0.80	3.12	0.183

Significant values in bold.
OR, odds ratio
CI, confidence interval
